# Large or multiple pseudocysts can impede or complicate the nonsurgical treatment of pancreatolithiasis

**DOI:** 10.20407/fmj.2022-011

**Published:** 2022-10-28

**Authors:** Satoshi Yamamoto, Kazuo Inui, Yoshiaki Katano, Hironao Miyoshi, Takashi Kobayashi, Yoshihiko Tachi

**Affiliations:** 1 Department of Gastroenterology, Fujita Health University Bantane Hospital, Nagoya, Aichi, Japan; 2 Department of Gastroenterology, Yamashita Hospital, Ichinomiya, Aichi, Japan; 3 Department of Gastroenterology, Fujita Health University Okazaki Medical Center, Okazaki, Aichi, Japan

**Keywords:** Chronic pancreatitis, Pancreatolithiasis, Pseudocyst, Extracorporeal shock wave lithotripsy

## Abstract

**Objectives:**

We aimed to determine when a coexisting pseudocyst was likely to complicate the nonsurgical treatment of pancreatolithiasis.

**Methods:**

We treated 165 patients with pancreatolithiasis nonsurgically between 1992 and 2020, including 21 with pseudocysts. Twelve patients had a single pseudocyst less than 60 mm in diameter. Pseudocysts in the other nine patients had diameters of at least 60 mm or were multiple. The locations of pseudocysts along the length of the pancreas varied from the area with stone involvement to the pancreatic tail. We compared the outcomes in these groups.

**Results:**

We found no significant differences in pain relief, stone clearance, stone recurrence, or the likelihood of adverse events between pseudocyst groups or between patients with vs without pseudocysts. However, 4 of 9 patients with large or multiple pseudocysts required transition to surgical treatment (44%) compared with 13 of 144 patients with pancreatolithiasis and no pseudocyst (9.0%) (*P*=0.006).

**Conclusions:**

Patients with smaller pseudocysts typically underwent nonsurgical stone clearance successfully with few adverse events, similar to findings in patients with pancreatolithiasis and no pseudocysts. Pancreatolithiasis complicated by large or multiple pseudocysts did not cause more adverse events but was more likely to require transition to surgery compared with pancreatolithiasis without pseudocysts. In patients with large or multiple pseudocysts, early transition to surgery should be considered when nonsurgical treatment is ineffective.

## Introduction

Chronic pancreatitis can lead to irreversible changes. Compromised pancreatic exocrine and endocrine function can eventually follow initial abdominal pain. Neither the nutritional consequences of exocrine dysfunction nor the manifestations of endocrine dysfunction are easily reversible, and patients do not recover spontaneously after treatment for chronic pancreatitis. The most common complication of chronic pancreatitis is pancreatic pseudocyst, which in turn can be complicated by infection, rupture, or bleeding. Another complication of chronic pancreatitis is pancreatolithiasis, which is usually treated nonsurgically. However, when an associated pseudocyst has a diameter of 60 mm or more, the need to transition to surgical treatment may be more likely than that when the pseudocyst measures less than 60 mm.^1^ To optimally prevent and treat such complications of chronic pancreatitis, many guidelines have been published by various medical and surgical societies.^2–6^ In 2020, the International Consensus Guidelines on Chronic Pancreatitis (ICGCP) were published as a synthesis of the preceding recommendations.^7^ As nonsurgical treatment for pancreatolithiasis, extracorporeal shock-wave lithotripsy (ESWL), first reported by Sauerbruch et al.,^8^ and endoscopic treatment are performed. When pancreatolithiasis and pseudocysts require treatment as concurrent complications of chronic pancreatitis, the pseudocysts must be considered as well as the stones. Nakagawa et al.^9^ first reported the occurrence of hemorrhage from a pseudoaneurysm in a pseudocyst following ESWL for pancreatolithiasis, concluding that performing ESWL for pancreatolithiasis in a patient with a pseudocyst requires particular care and consideration of risk.

We have performed ESWL for pancreatolithiasis, either alone or combined with endoscopic treatment, since 1992. Because nonsurgical treatment for pancreatolithiasis with a pseudocyst is difficult and few studies have analyzed high numbers of cases, in this study, we reported the outcomes of nonsurgical treatment of pancreatolithiasis in our patients with pseudocysts. We aimed to determine how the presence of pseudocysts affected the outcomes of nonsurgical treatment of pancreatolithiasis.

## Methods

### Patient characteristics

We retrospectively reviewed 165 patients with pancreatolithiasis who underwent nonsurgical treatment between 1992 and 2020. Among these 165 patients, 21 also had one or more pseudocysts. We compared patients with concurrent pancreatolithiasis and pseudocysts with those with pancreatolithiasis and no pseudocyst to determine whether the presence of a pseudocyst affected treatment outcomes or the rate of transition to surgery. Among the 21 patients with both pancreatolithiasis and pseudocysts, the median follow-up duration was 34 months (range, 0–117), and the median age was 60 (42–83) years. The male:female ratio was 9.5:1 (19 men, 2 women). The etiology of chronic pancreatitis was alcoholic in 14 patients and non-alcoholic in 7.

### Stone and pancreatic duct characteristics

Stones were solitary in 5 patients and multiple in 16. The mean stone size was 13 mm (standard deviation, 6 mm). Stone locations in the main pancreatic duct comprised the pancreatic head, body, and tail. The borders of these locations were defined in accordance with the tumor-node-metastasis classification.^10^ The stones in 14 patients were confined to one location, and in the other 7 patients, the stones were located in two or more areas. Stricture of the main pancreatic duct was defined as a high degree of stenosis (diameter: <2 mm), accompanied by dilation of the duct distal to the stenosis; eight patients had such a stricture (Table 1).

In 144 patients with pancreatolithiasis but no pseudocyst, the median follow-up duration was 31 (0–293) months. The median age was 57 (22–80) years, and the male:female ratio was 4.8:1 (119 men, 25 women). The etiology of chronic pancreatitis was alcoholic in 101 patients and non-alcoholic in 43. Stones were solitary in 74 patients and multiple in 70, and the mean stone size was 12 (standard deviation, 6) mm. Stones were limited to one location in 121 patients, while the remaining 23 patients had stones in two or more areas. Thirty-nine patients had a stricture of the main pancreatic duct, and patients with both pancreatolithiasis and pseudocysts more often had multiple stones compared with patients with pancreatolithiasis without a pseudocyst.

### Pseudocyst characteristics

Pseudocysts were solitary in 15 patients and multiple in 6. The mean diameter of the pseudocysts was 33 (standard deviation, 25) mm, and four patients had a pseudocyst with a diameter of 60 mm or more. The boundaries of the pseudocyst locations were defined in accordance with the tumor-node-metastasis classification,^10^ as for stone distribution. Nineteen patients (90%) had a pseudocyst in one location, while two patients (10%) had pseudocysts in more than one location (Table 1). The locations of pseudocysts along the length of the pancreas varied from the area with stone involvement to the pancreatic tail.

### Treatment

**Figure 1:**
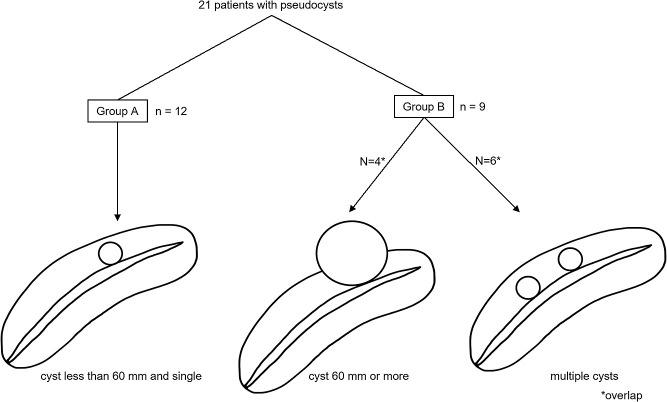
Classification of the 21 patients with pseudocysts

**Figure 2:**
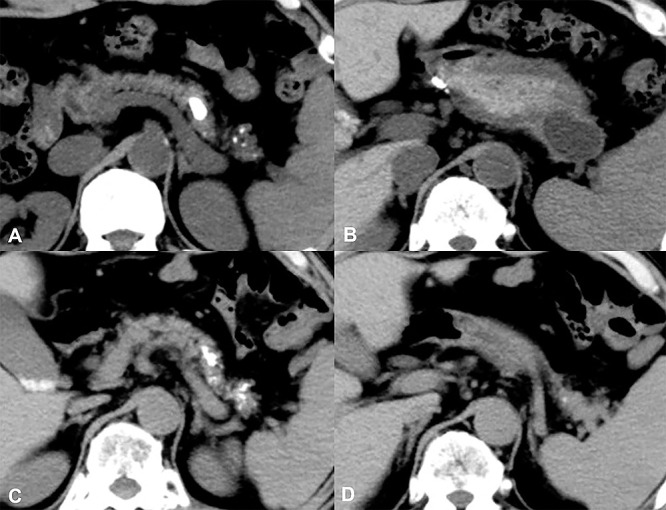
Clinical course in a patient in Group A with a pseudocyst (A) and (B): Before treatment, the size of the pseudocyst, which was located in the pancreatic tail, was 35 mm. The size of the stone, which was located in front of the cyst, was 14 mm. ESWL was performed five times. (C) and (D): After treatment, the stone was smaller, and no cyst was evident.

**Figure 3:**
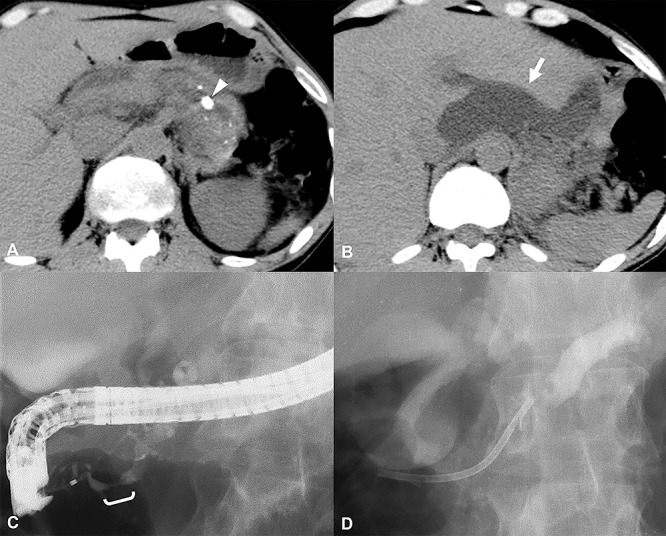
Clinical course of a patient in Group B with a pseudocyst (A) and (B): Before treatment, a stricture in the main pancreatic duct was present. The arrowhead shows a stone involving the main pancreatic duct within the body of the pancreas. The arrow shows the pseudocyst located behind the stone and near the liver. Percutaneous transhepatic pancreatic cyst drainage was performed. (C) and (D): No cyst was visualized after drainage was performed. A stent was placed in the head portion of the main pancreatic duct to resolve the stricture, and ESWL was performed to treat the pancreatolithiasis. However, the cyst recurred because the stricture in the main pancreatic duct had not resolved. Transition to surgery was required.

Patients with pseudocysts measuring 60 mm or more have been reported to require surgery more often than in those with smaller pseudocysts.^1^ For our analysis, we classified patients with a solitary pseudocyst measuring less than 60 mm as Group A, and those with pseudocysts measuring more than 60 mm or with multiple pseudocysts as Group B. Group A comprised 12 patients, and group B comprised 9 patients (Figure 1). No significant differences were evident between Groups A and B regarding age, sex, etiology, number of stones, stone location(s) limited to the head and/or body of the pancreas vs. location(s) including the tail, and the presence or absence of strictures involving the main pancreatic duct (Table 2). Pain was the main indication for nonsurgical treatment of pancreatolithiais.^7,11–16^ Additional indications were an impacted pancreatic stone causing dilation of the main pancreatic duct and compromised pancreatic function.^14,15,17^ Treatment for pancreatolithiasis with pseudocysts was similar to that for pancreatolithiasis with no pseudocyst. For patients in Group A, with a solitary pseudocyst measuring less than 60 mm, the stones were usually treated first (Figure 2). For Group B, with stones larger than 60 mm or with multiple pseudocysts, the pseudocysts were usually treated before the stones (Figure 3). For both stones and pseudocysts, resolution was defined as disappearance of the abnormality. Clearance was defined as removal of all detectable stones.

### Statistical analysis

We analyzed the outcomes of nonsurgical treatment for pancreatolithiasis in the following groups: no pseudocysts; with pseudocysts; with a solitary pseudocyst measuring <60 mm; and with pseudocysts measuring more than 60 mm or with multiple pseudocysts. We used the chi-squared test for univariate analysis and logistic regression analysis for multivariate analysis. Logistic regression analysis was used to calculate an adjusted odds ratio with 95% confidence interval. A P value of <0.01 was considered statistically significant. Statistical analysis was performed using SPSS V26.0 software (SPSS Statistics, version 26.0; IBM, Armonk, NY, USA).

The ethics committee at our institution approved this retrospective observational study.

## Results

### Outcomes of nonsurgical treatment for pancreatolithiasis

The overall stone clearance rate was 79% (130 of 165 patients). Among the 144 patients with no pseudocyst, 115 (80%) achieved stone clearance. Of the 21 patients with pseudocysts, 15 achieved stone clearance (71%). No significant difference was evident between these groups (*P*=0.550).

### Outcomes of nonsurgical treatment for pancreatolithiasis with pseudocysts on the basis of the patients’ characteristics

No significant differences in stone clearance rate or cyst resolution rate were seen for age, sex, etiology (alcoholic vs. non-alcoholic), number of stones, size of stones, distribution of stones (not including the tail of the pancreas vs. including the tail), or presence vs. absence of a stricture in the main pancreatic duct. However, transition to surgery was more frequent when pseudocysts were associated with a stone distribution that included the tail of the pancreas than when stone distribution did not include the tail (Table 3).

### Surgical transition rates according to pseudocyst presence and characteristics

Among 144 patients with pancreatolithiasis but no pseudocyst, 13 (9%) required surgery, while 5 of the 21 patients with both pancreatolithiasis and pseudocysts required surgery (24%), representing a somewhat increased rate (*P*=0.098) (Table 4). Among 9 patients with pseudocysts in Group B, 4 (44%) required surgery, while 13 of 144 patients with pancreatolithiasis but no pseudocyst required surgery (9.0%), representing a significantly higher surgical transition rate in Group B than that in patients with pancreatolithiasis without pseudocysts (*P*=0.006) (Table 4). The surgical transition rates did not differ significantly between Groups A and B.

### Outcomes of nonsurgical treatment on the basis of the features of the pseudocysts

All patients underwent ESWL. Among 12 patients in Group A, whose stones were usually treated by ESWL and/or endoscopic procedures before addressing the pseudocyst, 11 patients were managed with this approach. In the remaining patient, drainage of the pseudocyst via the major duodenal papilla was attempted before treatment for pancreatolithiasis. However, the guidewire could not reach the pseudocyst location because a portion of the main pancreatic duct between the papilla and the pseudocyst location was distorted and contained an impacted stone. Therefore, this patient underwent ESWL for pancreatolithiasis, followed by complete resolution of the stones and eventually, the pseudocyst.

Among the nine patients in Group B, in whom the pseudocyst was generally treated first, seven patients followed this sequence of treatment. Five of these patients underwent endoscopic nasopancreatic drainage via the major duodenal papilla, one patient underwent endoscopic ultrasound-guided pseudocyst drainage, and the other underwent percutaneous drainage. In six of the seven patients, pseudocyst treatment was technically successful. The patient whose treatment failed had multiple small pseudocysts (maximum size, 31 mm). In this patient, during a transpapillary endoscopic approach for drainage of a pseudocyst, the guidewire could not reach the targeted pseudocyst because of the presence of a stone in the main pancreatic duct. Two patients in Group B were treated for pancreatolithiasis first; both had multiple pseudocysts (maximum size, 16 mm). ESWL was performed for both patients before treatment of the pseudocysts, and ESWL succeeded in the removal of all stones from the main pancreatic duct. The pseudocysts resolved in one patient but not in the other.

Among the 12 patients in Group A (small solitary pseudocysts), 7 patients (58%) experienced cyst resolution, as did 4 of 9 patients in Group B (44%). These success rates did not differ significantly (Table 5). Among all 21 patients who had both pancreatolithiasis and pseudocysts, 4 patients experienced adverse events after nonsurgical treatment. Among these four patients, two in Group A and one in Group B developed mild pancreatitis after ESWL, and one patient in Group B developed mild pancreatitis after endoscopic nasopancreatic drainage. Adverse events in all patients were promptly relieved by conservative treatment. No significant differences between the groups regarding adverse events were detected in the multivariate analysis.

### Comparison of the outcomes of treatment in the pseudocyst groups and in the group with pancreatolithiasis without a pseudocyst

No differences were evident regarding the pain relief rate, stone disappearance rate, stone recurrence rate, or adverse event rate between the pancreatolithiasis without pseudocyst group, Group A, and Group B (Table 6). There were also no significant differences in the multivariate analysis.

### Details of the surgical transition cases

Of 21 patients who had pancreatolithiasis with a pseudocyst, 5 (1 in Group A and 4 in Group B) eventually required surgery (Table 7). The median time from the beginning of the treatment to surgery was 17 (0–38) months. The reason for surgery in two patients was a problematic stricture of the main pancreatic duct. Another patient who transitioned to surgery had a deformity of the main pancreatic duct that precluded endoscopic intervention, resulting in acute pancreatitis that recurred after each ESWL procedure. Another patient requiring surgery achieved cyst reduction. However, 1 month after nonsurgical treatment, hemorrhage into the pseudocyst occurred; this resolved spontaneously but recurred repeatedly. In this patient, a pseudoaneurysm within the pseudocyst was not recognized, and we attributed the hemorrhage to a fragile, chronically inflamed cyst wall. One patient required surgery because pancreatolithiasis was difficult to treat owing to stricture of the bile duct resulting from chronic inflammation.

## Discussion

When conservative treatment does not effectively resolve abdominal pain from chronic pancreatitis because of a stone in the main pancreatic duct, the ICGCP recommends ESWL as the first-line nonsurgical treatment. ESWL is often followed by endoscopic treatment to remove small stones and stone fragments. The ICGCP also states that either nonsurgical treatment or surgery can be performed to treat pseudocysts causing symptoms.^7^ Aljarabah et al.^17^ systematically reviewed endoscopic and surgical treatments for pseudocysts. Among 569 patients who underwent endoscopic drainage, 80.8% had successful outcomes, and treatment was successful in 98.3% of 118 patients whose drainage procedure was laparoscopic. No significant difference in the likelihood of a favorable outcome was evident between endoscopic vs surgical treatment.

Unfortunately, guidelines and consensus statements have not considered ESWL as a treatment for pancreatolithiasis in the specific context of coexisting pseudocysts.^18^ One cause of pseudocysts is obstruction of the main pancreatic duct causing pancreatic ductal hypertension behind the obstruction, as can occur with a pancreatic stone in a patient with chronic pancreatitis.^19^ A pseudocyst caused by pancreatolithiasis can be effectively treated by removing the stone using ESWL combined with endoscopic treatment. However, the safety and efficacy of this strategy are currently unclear. Simple drainage might be successful for an isolated pseudocyst; however, if a main pancreatic duct stone or stricture is present, and it obstructs outflow of pancreatic juice, resolution of the pseudocyst is not possible.

Many studies have evaluated the outcomes of nonsurgical treatments for pancreatolithiasis or pseudocysts; however, we know of only one previous report dealing with nonsurgical treatment outcomes in a high number of patients with both pancreatolithiasis and pseudocysts.^18^ Among the 59 such patients described by Li et al., 67.24% achieved stone clearance, compared with 83.17% of 790 patients with pancreatolithiasis and no pseudocyst; the difference between stone clearance rates was not statistically significant.^18^ In our study, 15 of 21 patients with both pancreatolithiasis and pseudocysts (71%) achieved stone clearance, compared with 115 of 144 patients who had pancreatolithiasis without pseudocysts (80%). The outcomes of treatment for pancreatolithiasis were good in both of these groups.

When we compared the treatment results for patients with pancreatolithiasis and no pseudocyst, patients with a single pseudocyst measuring less than 60 mm in diameter, and patients with pseudocysts measuring more than 60 mm or with multiple pseudocysts, no significant differences were evident regarding the pain relief rate, stone clearance rate, stone recurrence rate, cyst reduction rate, cyst resolution rate, cyst recurrence rate, or adverse event rate. However, pancreatolithiasis with pseudocysts that were multiple or measured 60 mm or more had a significantly higher surgical transition rate than that with pancreatolithiasis without a pseudocyst. While surgery is invasive by nature, it is required when nonsurgical treatment is unsuccessful or impossible.^20^ The most compelling reason underlying the need for surgery in Group B was stricture of the main pancreatic duct and/or bile duct as a complication of chronic pancreatitis (Table 7). We also suspect that pancreatolithiasis with concurrent large or multiple pseudocysts could be an early unfavorable indicator in chronic pancreatitis. This was also the most important reason why our Group B patients were more likely to require surgery compared with the patients with stones but no pseudocysts. Treatment for pancreatolithiasis with pseudocysts can begin nonsurgically. However, when pseudocysts are large or multiple, timely surgical transition should be considered when a patient’s course following nonsurgical treatment fails to improve.

Of Li et al.’s 59 patients with both pancreatolithiasis and pseudocysts, 7 (11.86%) experienced adverse events. No rupture of a pseudocyst or bleeding into a pseudocyst occurred. However, one of the seven patients had enlargement of a pseudocyst accompanied by pain, representing a moderate to severe adverse event; eventually the patient required surgery. Among the 790 patients in the study who had pancreatolithiasis but no pseudocysts, 98 experienced adverse events (12.41%); however, the rate was not significantly different from that of patients with both pancreatolithiasis and pseudocysts.^18^

ESWL equipment delivers shock waves to pancreatic stones through a water-filled cushion. Li et al.^18^ suggested that because a pseudocyst is filled with fluid, pseudocyst contents might absorb only limited energy from the shock waves, as with other aqueous wave transmission media. This should increase the safety of ESWL when used for stones near a pseudocyst, decreasing the likelihood of pseudocyst rupture during ESWL for pancreatolithiasis.

Nakagawa et al.^9^ reported a case in which hemorrhage occurred within a pseudocyst following ESWL. The authors described the use of ESWL to treat multiple pancreatic stones in a patient with a pseudocyst measuring 30 mm in diameter in the pancreatic tail; a hemorrhagic pseudoaneurysm resulted, and the pseudocyst enlarged. Pancreatic pseudoaneurysms typically result from pancreatic digestive enzymes in the fluid within a pseudocyst.^21^ However, Nakagawa et al. concluded that the occurrence of pseudoaneurysm in their patient after ESWL most likely resulted from the treatment itself.

Among 12 patients in our Group A, 2 patients experienced adverse events (17%), while 2 of 9 patients in Group B experienced adverse events (22%). No significant difference in the frequency of adverse events was evident between these groups or between either group and patients with pancreatolithiasis and no pseudocyst (14 of 144 patients (9.7%)). Among the patients with pancreatolithiasis and a pseudocyst, all patients with adverse events developed mild acute pancreatitis; no severe adverse events occurred. Because pseudoaneurysms can develop spontaneously from a pseudocyst, contrast-enhanced computed tomography should be performed for all patients with both stones and pseudocysts, before and after nonsurgical treatment, whenever possible. Doing so should increase safety by detecting pseudoaneurysms prior to stone treatment.

Pseudocysts with stones located in the tail of the pancreas had a higher surgical transition rate, in this study. However, no significant difference was evident regarding the surgical transition rate between Group A and Group B. Only 1 of 12 patients in Group A required surgery; this patient experienced acute pancreatitis after each ESWL treatment. Although we tried to place a stent in the main pancreatic duct to prevent these recurrences, placement proved difficult because of deformity of the main pancreatic duct. Therefore, this patient was transitioned to surgery because of the indication of failure or impossibility of nonsurgical treatment.^20^ Rosso et al.^22^ suggested the following additional indications for surgery: numerous or complicated strictures of the main pancreatic duct; complex underlying pathologies, such as an inflammatory tumor of the pancreatic head; stricture of the bile duct; multiple pseudocysts; pseudocyst in the pancreatic tail; and suspicion of a neoplastic cyst. One of our patients in Group B, who had pancreatolithiasis with a pseudocyst and stricture of the bile duct, was transitioned to surgery because of difficulty treating both the pancreatolithiasis and the pseudocyst.

In our study, pancreatolithiasis with multiple or large pseudocysts was associated with a significantly higher surgical transition rate than that in patients with pancreatolithiasis without a pseudocyst. If nonsurgical treatment is ineffective for pancreatolithiasis accompanied by multiple or large pseudocysts, surgical options should be considered expeditiously. However, factors favoring transfer to surgical management of pancreatolithiasis with pseudocysts typically interact in a complex manner. As a result, the conclusions of a study such as ours are not absolute, and investigations involving larger numbers of patients are needed.

In conclusion, in the nonsurgical treatment of pancreatolithiasis with a pseudocyst, our stone clearance rate was favorable, and adverse events were uncommon, with a similar rate to that in patients with pancreatolithiasis without a pseudocyst. We consider that nonsurgical treatment, including with ESWL, is safe and effective for patients with pancreatolithiasis accompanied by pseudocysts. However, our patients with pseudocysts coexisting with stones located in the tail of the pancreas had a higher surgical transition rate. Nonsurgical treatment for pancreatolithiasis in the presence of multiple or large pseudocysts was as safe as that for pancreatolithiasis without pseudocysts. However, patients with pancreatolithiasis with large (60 mm or more) or multiple pseudocysts eventually required surgery more often compared with patients with pancreatolithiasis without a pseudocyst. If nonsurgical treatment of pancreatolithiasis, including with ESWL, is not successful when lithiasis involves the tail of the pancreas and when large or multiple pseudocysts are present, surgical intervention must be considered.

## Figures and Tables

**Table1:** Features of Pancreatolithiasis in 165 Patients

	With Pseudocysts, n (%), (n=21)	Without Pseudocysts, n (%), (n=144)	*P*
Patient characteristics			
Etiology			
Alcoholic	14 (67)	101 (70)	0.945
Non-alcoholic	7 (33)	43 (30)	
Number of stones			
Single	5 (24)	74 (51)	0.033
Multiple	16 (76)	70 (49)	
Size of the stones			
<10 mm	5 (24)	52 (36)	0.389
≥10 mm	16 (76)	92 (64)	
Distribution of the stones			
One area	14 (67)	121 (84)	0.104
Two or more areas	7 (33)	23 (16)	
Stricture in the main pancreatic duct			
Absence	13 (62)	105 (73)	0.432
Presence	8 (38)	39 (27)	
Pseudocyst characteristics			
Cyst Number			
Single	15 (71)		
Multiple	6 (29)		
Cyst Size			
<60 mm	17 (81)		
≥60 mm	4 (19)		
Locularity			
Unilocular	18 (86)		
Multilocular	3 (14)		
Cyst distribution			
One area	19 (90)		
Two or more areas	2 (10)		

**Table2:** Characteristics of the Patients in Groups A and B

Patient Characteristic	Group A, n (%), (n=12)	Group B, n (%), (n=9)	*P*
Age, y			
<65	4 (33)	8 (89)	0.036
≥65	8 (67)	1 (11)	
Sex			
Male	10 (83)	9 (100)	0.592
Female	2 (17)	0 (0)	
Etiology			
Alcoholic	6 (50)	1 (11)	0.161
Non-alcoholic	6 (50)	8 (89)	
Number of Stones			
Single	4 (33)	1 (11)	0.506
Multiple	8 (67)	8 (89)	
Size of the Stones			
<10 mm	5 (42)	0 (0)	0.089
≥10 mm	7 (58)	9 (100)	
Distribution of the Stones			
Head to body	11 (92)	6 (67)	0.378
Including tail	1 (8)	3 (33)	
Stricture in the Main Pancreatic Duct			
Present	8 (67)	5 (56)	0.948
Absent	4 (33)	4 (44)	

**Table3:** Characteristics of Pancreatolithiasis with Pseudocysts: Influences on Stone Clearance, Cyst Resolution, and Surgical Transition After Nonsurgical Treatment (N=21)

Characteristic	Stone Clearance Rate, %	*P*	Cyst Resolution Rate, %	*P*	Surgical Transition Rate, %	*P*
Age, y						
<65 (n=12)	67	0.944	42	0.488	42	0.089
≥65 (n=9)	78		67		0	
Sex						
Male (n=19)	74	1.000	58	0.415	26	1.000
Female (n=2)	50		0		0	
Etiology						
Alcoholic (n=14)	71	1.000	64	0.280	14	0.856
Non-alcoholic (n=7)	71		29		29	
Number of Pancreatic Stones						
Single (n=5)	100	0.292	60	1.000	0	0.406
Multiple (n=16)	63		50		31	
Size of the Stones, mm						
<10 (n=5)	80	1.000	20	0.251	20	1.000
≥10 (n=16)	69		63		25	
Distribution of the Stones						
Head to body (n=17)	71	1.000	53	1.000	12	0.043
Including tail (n=4)	75		50		75	
Stricture of the Main Pancreatic Duct						
Present (n=8)	63	0.831	54	1.000	50	0.092
Absent (n=13)	77		50		7.7	

**Table4:** Surgical Transition Rate According to Pseudocyst Presence and Characteristics

Pseudocyst	Surgical Transition Rate, n/N (%)
Absent, n=144	13/144 (9.0) *
Present, n=21	5/21 (24)
Group A, n=12	1/12 (8.3)
Group B, n=9	4/9 (44) *

*P=0.006

**Table5:** Treatment Results in 21 Patients with Pancreatolithiasis and Pseudocysts

	Group A, n/N (%)	Group B, n/N (%)	*P*
Pain Relief	6/9 (67)	7/9 (78)	1.000
Stone Clearance	9/12 (75)	6/9 (67)	1.000
Stone Recurrence	4/9 (44)	3/6 (50)	1.000
Cyst Reduction	9/12 (75)	8/9 (89)	0.810
Cyst Resolution	7/12 (58)	4/9 (44)	0.850
Cyst Recurrence	0/7 (0)	1/4 (25)	0.766
Adverse Events	2/12 (17)	2/9 (22)	1.000

**Table6:** Treatment Outcomes for Pancreatolithiasis According to the Presence of Pseudocysts

Pseudocysts	Pain Relief, n/N (%)	Stone Clearance, n/N (%)	Stone Recurrence, n/N (%)	Adverse Events, n/N (%)
Absent, n=144	104/112 (93)	115/144 (80)	43/115 (37)	14/144 (9.7)
Present, n=21				
Group A, n=12	6/9 (67)	9/12 (75)	4/9 (44)	2/12 (17)
Group B, n=9	7/9 (78)	6/9 (67)	3/6 (50)	2/9 (22)

**Table7:** Reasons Why Five Patients with Chronic Pancreatitis Required Surgery

Case	Group	Number of ESWLs Performed	Number of Endoscopic Treatments	Interval to Surgery, mo	Reason
1	A	2	2	0	deformation of the MPD
2	B	5	1	20	hemorrhage into the pseudocyst
3	B	3	6	17	stricture of the MPD
4	B	10	2	38	stricture of the CBD
5	B	6	3	2	stricture of the MPD

ESWL, Extracorporeal Shock Wave Lithotripsy; MPD, Main Pancreatic Duct; CBD, Common Bile Duct Case 1, pancreatoduodenectomy; Case 2, distal pancreatectomy; Case 3, Frey procedure; Case 4, distal pancreatectomy; Case 5, cholangiojejunostomy and lateral pancreaticojejunostomy
